# A nanobiosensor based on graphene oxide and DNA binding dye for multi-microRNAs detection

**DOI:** 10.1042/BSR20181404

**Published:** 2019-12-13

**Authors:** Mahdi Rahaie, Saman Khayat Noroozi

**Affiliations:** Department of Life Science Engineering, Faculty of New Sciences and Technologies, University of Tehran, Tehran 1439957131, Iran

**Keywords:** Alzheimer’s disease, Early Detection, Fluorescence, microRNA, Nanobiosensor

## Abstract

Multiplex assays for detection of biomarkers, provide advantageous analyses of different factors related to diagnoses of diseases. The Alzheimer’s disease (AD) is one of the most common disease in old people in societies which is increasing, significantly. A group of microRNAs (miRNAs) play an important role in developing the disease which can be considered as early stage biomarkers. Since, selective, sensitive, simple and rapid method for detection of these miRNAs in a single test is critical for early diagnosis and efficient therapy of the disease, herein, we report a sensitive fluorescence assay based on enzyme-free and isothermal hybridization chain reaction with SYBR Green and graphene oxide (GOX) for early detection of miR-137 and miR-142, as two Alzheimer’s biomarkers. Fluorescence spectrophotometry based on SYBR Green signal and GOX as the fluorescence quencher was used for detection and quantification of targets’ miRNAs and change in fluorescence intensity due to absence and presence of the targets was measured. The limit of detection in the newly designed nanobiosensor was achieved as 82 pM with a sensitive detection of the miRNAs from 0.05 to 5 nM, that is critical for detecting the biomarkers. Given the real range of concentrations of miRNAs in blood (from nanomolar to femtomolar values), the method holds great promise in dual and multiple targets detection due to its sensitivity, rapidness, inexpensive and specificity which provides a convenient detection method of Alzheimer’s in early stage.

## Introduction

Alzheimer’s disease (AD) is a progressive neurodegenerative disease, which in addition to huge economic impact ($818 billion worldwide in 2015), creates a list of abnormalities such as loss of memory, cognitive impairments and incidence of behavior and personality. Despite extensive research into AD, there is still not a drug or agent/s that can delay or prevent AD progression completely [1].

Generally, the diagnostic biomarkers are associated with the detection of disease, whereas prognoses of biomarkers could estimate the response to treatment. The presence or absence or changes in the ratio of the specific biomarkers in a cell, tissue or body fluids often indicate the disease development such as cancer [2]. The early detection and precise diagnosis of the disease is the most promising approach to accelerate healing processes or to improve therapy of patients [3]. Investigation of the biomarkers ratio can help in early diagnosis and disease progression monitoring [2]. There is no doubt that their exact identification in early stage is crucial for good treatment and reducing cost for Alzheimer’s patients [4]. MicroRNAs (miRNAs) are small noncoding RNAs with a length from 18 to 22–23 nucleotides that act in post-transcriptional modifications related to their target genes in different eukaryotic and prokaryotic organisms [5,6]. Mature miRNAs regulate gene expression by catalyzing the cleavage of mRNA and incorporation into RNA-induced silencing complex (RISC) where they interact with complementary sites on target mRNA and then, downstream regulation of its expression [7–9]. Especially, some miRNA expression patterns are associated with some malignant diseases such as liver-related diseases, cardiovascular diseases, cancers and neurodegenerative diseases such as Alzheimer, Multiple sclerosis (MS) and Parkinson. Therefore, miRNAs are considered as potential biomarkers specific to corresponding tissues or diseases [10]. Several miRNAs are well-studied in AD including mir-137 [11], mir-142-5p [12], mir-181c [13] and mir-29a/b [14,15] and expression levels some of them are altered in the diseased state.

The detection of miRNA is a challenge due to its instability, short sequence, trace amounts and the complex interferences from biological samples miRNA [7,9]. Conventional methods, such as real-time RT-Polymerase chain reaction (PCR), microarray analysis and Northern blotting, offer high accuracy and sensitivity for miRNAs measurement [5,16]. However, most of these methods require time-consuming sample pretreatment, tedious and complicated procedures and harsh experimental conditions and high experimental cost, as well [17–21]. These issues dramatically restrict their further practical applications. Accordingly, there is a high request to develop a simple, rapid and cost-effective approach for precise detection of miRNA. Nowadays, the achievements in nanotechnology, nanoparticles and fluorescence-based biosensors have aroused great interests, due to high sensitivity and selectivity for target analysis [22–24]. Also, a critical subject for design and fabrication of a device for detection of biomarkers is multiple and simple detection in every test. In fact, multiple detection provides more valid diagnoses or prognoses of diseases.

Unlike common biosensors based on enzymes or antibodies, DNA-based biosensors having high sensitivity can be prepared with high assembly efficiency and low cost. The use of new nanomaterials has promoted the development of DNA biosensors with the goal of simple, selective and inexpensive detection for targets analysis [27]. The DNA-based biosensors which utilize DNA strands as probes for sensing targets and the processes of target recognition occurs in two ways including: (1) Hybridization of complementary probes with nucleotide targets, including DNA mutation and SNP detection (nearly most of sensors use this approach), (2) association of probes with targets or subunits related to specific properties of targets such GC-rich regions as host for silver nanoclusters (AGNCs) [25,26].

Graphene is a new emerging nanomaterial in the last decade which has unique electronic, adsorption and fluorescence properties. Hence, different kinds of graphene including Graphene oxide (GOX), reduced GOX (rGO) and Graphene ribbon (GR) have been used as key elements of biosensors for detecting assays [28,29,30,31–34]. For example, Graphenes can quench fluorescence of DNA intercalating dye very perfectly, thus it can be used as a broad-spectrum fluorescence quenching agent, and can be made as essential elements of DNA-based optical sensors [2,34,35].

SYBR Green I similar to Eva Green is one of the most sensitive stains available for detecting dsDNA, which has weak background fluorescence in the absence of DNA, and shows less attachment for ssDNA but high affinity for dsDNA and has large fluorescence intensity upon dsDNA binding [35].

PCR is the main approach for the amplification of DNA molecules; because the DNA self-polymerization can be progressed selectively and specifically without any polymerase enzyme. In the reaction, there are two hairpin sequences with sticky ends (P1 and P2) that are complementary to target sequence. Two stable stem-loop nucleotide structures coexist in solution until the addition of initiator strands’ operators a cascade of hybridization events that provide nicked double helix [36,37].

Although, these currently available DNA biosensors have provided high promise, but in our opinion, there still remains some point of view for improvement: (1) multiple targets detection simultaneously in each test can help to get precise view about the disease, (2) utilization of new methods to decrease the limit of detection, specially for some biomarkers such as miRNAs, (3) new output signals to fulfill the detection style with decreasing noise.

As an extension of the previous works of our group [36–38], herein, we report the design and fabrication of a nanobiosensor based on a fluorescence approach for simultaneous detection of two miRNA biomarkers involved in AD simultaneously, for early and simple diagnosis of the disease in patients. These biomarkers have different expressions in Alzheimer’s, so that one is down-regulated (miR-137) and another one is up-regulated (miR-142) and hence, it provides a confident method to clinicians and analysts for monitoring the diseases’ initiation and progression up on the efficient miRNA biomarkers, such as Alzheimer’s, MS, cancer etc.

## Experimental

### Reagents and chemicals

All chemicals were purchased from commercial sources and used without further purification. Na_2_HPO_4_, NaCl, Tris/HCl and EDTA were purchased in analytical grade from Sigma and Merck companies. SYBR Green I was obtained from GeneAll, (South Korea). GOX was a gift. All oligonucleotides used in the present study including Synthetic Target miRNAs, miR-137 (5′-TTATTGCTTAAGAATACGCGTAG-3′) and miR-142 (5′-TGTAGTGTTTCCTACTTTATGGA-3′), P1 (5′-CTTAAGACAATAACTACGCGTATTCTTAAGCAATAA-3′), P2 (5′-TTATTGTCTTAAGTCCATAAAGTAGGAAACACTACACTTAAGA-3′), P3 (5′-GATGCGCATAAGAATTCGTTATTTCTTAAG-3′) and non-Target sequence (5′-GTCCAGTTTTCCCAGGAATCCCT-3′) and sequence with two polymorphism bases (5′- TGATTGCTTAATAATACGCGTAG-3′) were synthesized by Macrogen Inc. (Seoul, Republic of Korea) in HPLC purified lyophilized powder and then dissolved in ultrapure water or buffer. All stock solutions were stored at −20°C. For HCR system, concentrated oligos stock solutions were prepared in SPSC buffer (50 mM Na_2_HPO_4_, 1 M NaCl (pH 7.5)) and followed by dilution according to reaction conditions. Other used solutions were prepared in deionized water.

### Apparatus

Fluorescence measurements were done by a Varian Cary Eclipse Fluorescence Spectrophotometer (Varian, Australia). UV-Vis absorption spectra were recorded with a NanoDrop 2000c Spectrophotometer (Thermo Scientific Inc., U.S.A.). The electrophoresis system consisted of a steady voltage power supply and horizontal electrophoresis tank (Pars Gene, Iran). Gel electrophoresis images were analyzed using a gel documentation system (Quantum ST4, Fisher Biotec Pty Ltd, Australia).

### Amplification by HCR and gel electrophoresis

For performance of HCR reaction, oligonucleotides stock solutions (100 μM) were prepared in SPSC buffer which were then diluted to reaction-optimized conditions. The stock solutions of probes were heated to 95°C for 3 min and then it was allowed to cool on ice for 1 h before use. Different concentrations of target miRNAs were incubated with 50 nM of each P_1_, P_2_ and P_3_ at 37°C for 15 min. A 1.5% agarose gel was prepared using 1× TAE buffer (40 mM Tris-base, 2.0 mM EDTA with pH 8.0). To confirm the HCR reaction, the Gel Red™ (Biotium Inc., U.S.A.) was used for visualization of HCR products on gel on mixing with samples (10 μl HCR products). The gel was run at 80 V for 60 min in 1× TBE buffer. The gel was exposed to UV light using the gel documentation system.

### miRNA detection by the nanobiosensor

For detection of both miR-137 and miR-142 to gather, first P_1_, P_2_ and P_3_ probes were heated to 95°C for 3 min and then it was allowed to cool on ice for 15 min before use. Different concentrations of miRNAs were mixed with probes in an SPSC buffer solution and were incubated at 37°C for 2 h. Subsequently, the fluorescence spectra were measured in a 160-μl quartz cuvette. Fluorescence intensity was recorded using a Fluorescence Spectrophotometer with excitation and emission wavelengths of 490 and 520nm, respectively. The specificity of our method was investigated by evaluating three replicate tests of the nanobiosensor response to different kinds of targets including perfect complementary target, double base pair mismatch and non-target miRNA, all with the same concentration (0.5 nM). For testing the nanobiosensor function in serum, the blood sample was taken from a healthy people and collected in a tube containing EDTA (0.01% v/v of 0.5 M Na_2_EDTA (pH 8.0)) to a final concentration of 10 mM and gently inverted several times. In follow, it was centrifuged at 4°C twice at 3000 rpm for 4 min and serum was isolated. The serum was diluted with water (1/50 ratio) and then, different concentrations of target miRNAs were made with serum media.

## Results and discussion

### The principle of label-free and enzyme-free ratiometric fluorescence nanobiosensor

**Scheme 1:**
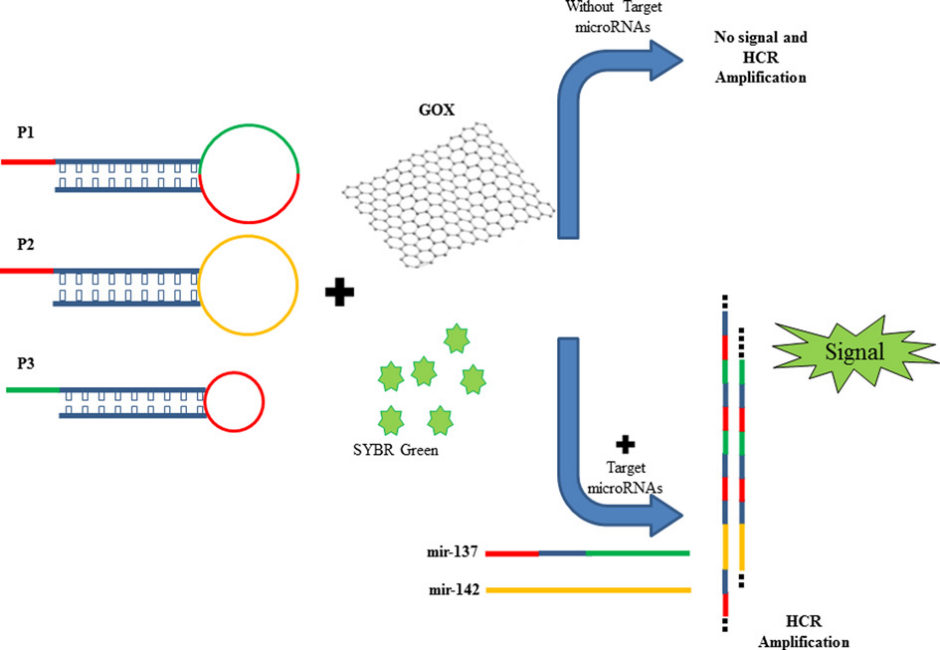
A schematic of fluorescent DNA-SYBR green modulated with HCR in the presence of target miRNAs

The HCR method as an enzyme-free amplification method was designed to induce a hybridization of the hairpins in the presence of target strands (miR-137 and miR-142). As shown in Scheme 1, the nucleic acid machine consists of two moieties, including detection and amplification. We used three complementary oligonucleotides (P1, P2 and P3) to form nicked double-helix molecules which are in separate from each other and as hairpin structure in absence of targets and closed in presence of target microR (miR-137 and miR-145) at room temperature. The target miRNAs acts as an initiator strand and make possibility to happen a cascade hybridization event which eventually leads to the nicked double-helix construction. Amplification of the initiator recognition event is continued until the supply of P1, P2 or P3 is finished (Scheme 1). ssDNA (probes and target miRNAs) due to flexibility, to be uncoiled and accessibility of its bases can be easily absorbed on GOX surface. On the opposite, double-stranded DNA (the nicked double helix resulting from HCR) is stable and the exposed negative charges in its backbone makes strong repulsion between dsDNA and GOX which causes weak binding between them and then it could not quench (through fluorescence resonance energy transfer (FRET) mechanism) intercalated SYBR Green Dye into dsDNA. As a consequence, fluorescent intensity in 520 nm increases, dramatically. This method is simple which does not require labeling and enzymatic reaction, modification or binding other fluorescent tags, and then it provides a highly sensitive and selective detection of diseases, miRNA biomarkers and other nucleic acids (such cell-free DNAs, viruses) and in body fluids such as blood. The average molecular weight of the resulting polymers is inversely related to the initiators concentration and time period of HCR, partly. Afterward, the strong repulsion between dsDNA and negatively charged GOX makes their binding, weak, which cannot inhibit SYBR green quenching.

### Optimization and validation of HCR and experimental conditions

**Figure 1:**
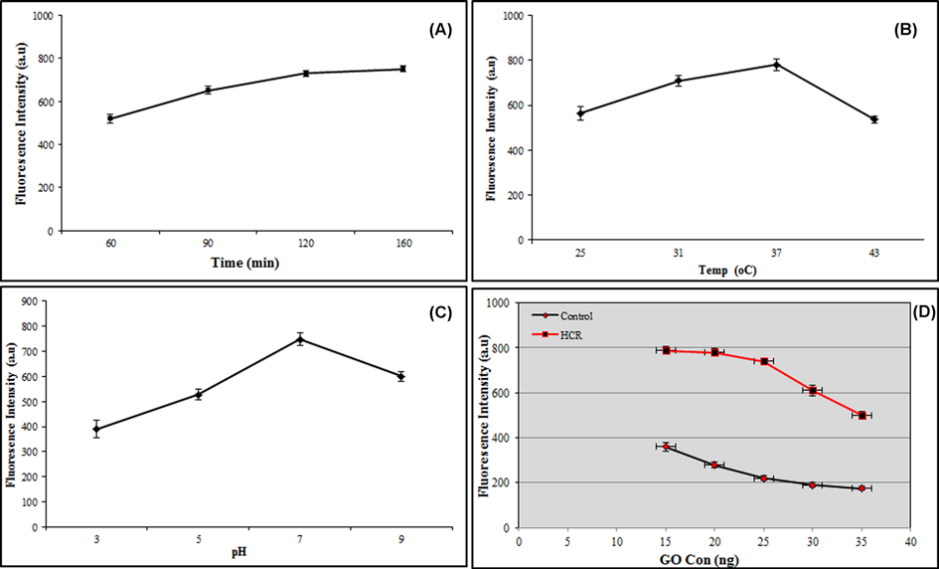
Parameter optimization of the nanobiosensor (**A**) Time, (**B**) Temperature, (**C**) pH and (**D**) GOX concentration.

To get an efficient HCR system, in addition to accurate designing of oligonucleotide probes, different parameters including temperature (25, 31, 37 and 43°C), pH (3, 5, 7 and 9), time (60, 90, 120 and 160 min), GOX concentrations (15, 20, 25, 30 and 35 μg/ml) and various buffers (phosphate, PBS, SPSC, Tris/HCl and ultra-pure water) were investigated in our protocols. It was found the ambient temperature for efficient HCR, the best pH for getting most stability of fluorescent by SYBR green, least consumed time for preparation the reagents and HCR before fluorescent assays, the best concentration of GOX for quenching of SYBR Green and getting the most signal to noise ratio of the nanobiosensor and the perfect media for the reactions were 37°C, pH 7, 2 h, 25 μg/ml and PBS buffer, respectively (Figure 1).

**Figure 2:**
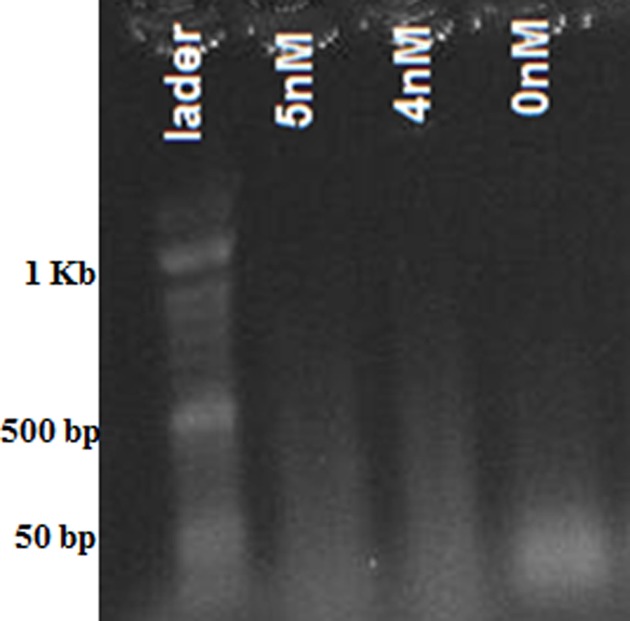
The gel electrophoresis of HCR products

The result of HCR reaction with different concentrations of target miRNAs was confirmed by agarose gel electrophoresis (Figure 2). Electrophoretic bands in the presence of various concentrations of target miRNAs are made which are related to the HCR reaction that creates polymers with different lengths. In the absence of miRNAs, no bands (smear) corresponded to the HCR reaction was seen (expect for the band of used oligonucleotides which are less than 50 bp).

**Figure 3:**
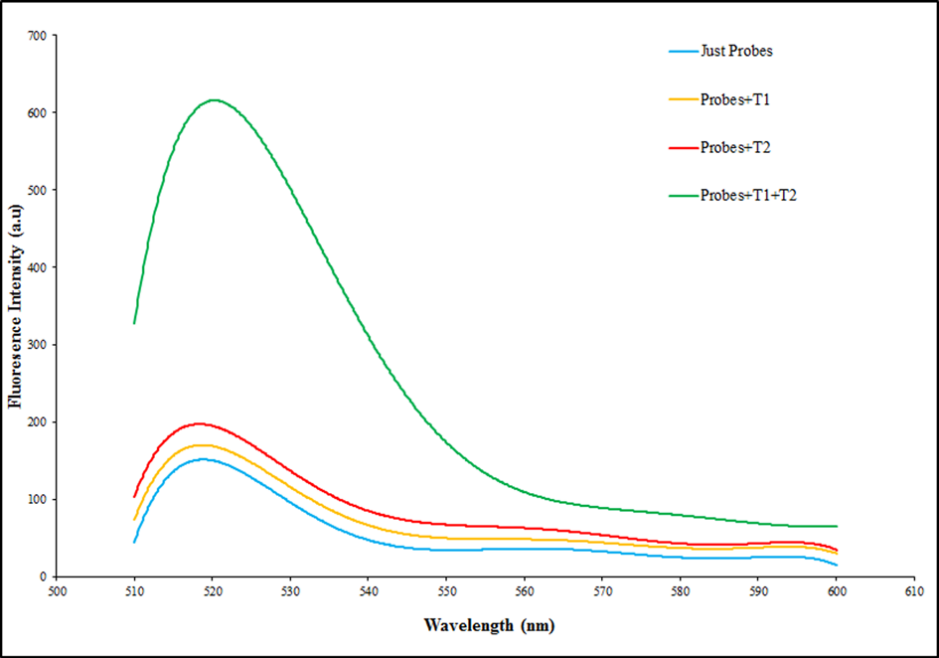
Nanobiosensor function Analysis Confirmation of nanobiosensor function with, a (blue): Probes (50 nM) without targets, b (yellow): Probes (50 nM) with miR-137 (5 nM), c (red): Probes (50 nM) with miR-142 (50 nM) and d (green): Probes (50 nM) with miR-137 (2 nM) and miR-142 (50 nM).

To demonstrate the feasibility and function of the present label-free and enzyme-free ratiometric fluorescence nanobiosensor, four tests was done with probes and targets in presence of SYBR green and then adding GOX, including 1-probes (50 nM) without targets, 2- probes (50 nM) with miR-137 (5 nM), 3-probes (50 nM) with miR-142 (50 nM) and 4-probes (50 nM) with miR-137 (2 nM) and miR-142 (50 nM). As shown in Figure 3, the fluorescence intensity with absence of targets is so much less compared with presence of targets due to quenching of free SYBR green in reaction by GOX. In fact, HCR occurrence and in follow, long chain nicked double stranded formation due to initiators (target miRNAs) is the reason of more fluorescence intensity.

### Ratiometric fluorescence analysis and sensitivity assessment of nanobiosensor

**Figure 4:**
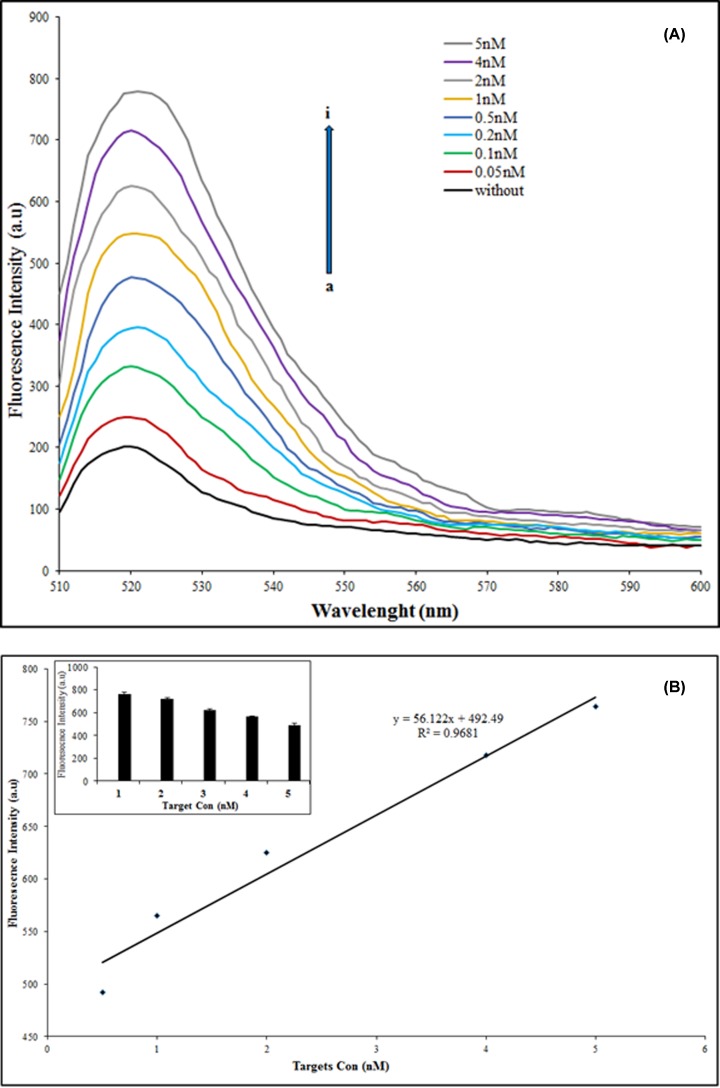
Sensitivity assessment of nanobiosensor (**A**) Fluorescence emission spectra in the presence of different concentrations of miRNA from 0.05 to 5 nM (a→i). (**B**) The linear relationship (R^2^ = 0.99) between the drawdown fluorescence intensity, in the presence of different concentrations of miRNA (0.5–5 nM). The inset graph shows the same results as histograms. The excitation wavelength was 490 nm.

To investigate the sensitivity and quantitative behavior (dependence of the fluorescence intensity on the different amounts of target miRNAs) of the proposed nanobiosensor, quantitative analysis of target miRNAs was performed by monitoring the fluorescence spectra of SYBR Green upon incubation with various concentrations of target miRNAs. As shown in Figure 4A, with the increasing concentration of target miRNAs used for hybridization in HCR, fluorescent emission intensity increases in a linear mode. The plot of the ascending fluorescence intensity vs. the concentration of target miRNAs shows a linear relationship, y = 56.122x + 492.49, R^2^ = 0.9681, in the range from 0.5 to 5 nM of miR-137 and a constant concentration on miR-142 (50 nM) (Figure 4B). Figure 4B compared with Figure 4A shows even though the nanobiosensor can detect less than 0.5 nM of targets but in the range of 0.5–5 nM has the most significant and creditable (R^2^ = 0.9681) detection. These results confirmed the principle of the nanobiosensor for simultaneous detection of double target miRNAs with different concentrations (miR-137 and miR-142 as down and up-regulated biomarkers, respectively) in solution. It was found a limit of detection, 0.082 nM for each target miRNA is achievably, readily.

Although a few previous works [37,39,40] have reported low sensitivity for their detection method, but our nanobiosensor due to use of HCR, SYBR green and GOX simultaneously and detection of more than one target molecule with a single nanostructure at the same time suggests a sensitive, favorable and more simplify technique for multiplex recognition of miRNA targets compared with other DNA/RNA sensors [41].

### Specificity of nanobiosensor

**Figure 5:**
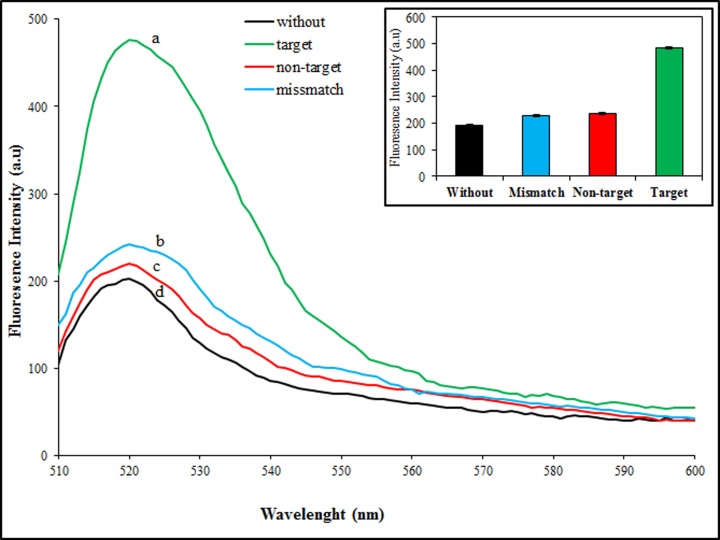
Specificity assessment of nanobiosensor Fluorescence emission spectra of the nanostructure in the present of complementary targets (a), two base mismatched targets (b), non-complementary targets (c) and without targets (d). Inset, the fluorescence emission efficiency adding with different targets.The excitation wavelength was 490 nm.

Specification in the action of a sensor is an important criterion for every detection system. For analysis of our method selectivity and getting accuracy, we investigated fluorescence intensity shift analysis in the presence of complementary targets (both), two-base mismatched and non-complementary targets (regard to only miR-137 sequence) with three replicates. The sample without target was used as a negative control. As depicted in Figure 5, changes in fluorescence intensity are too much less and negligible in the presence of mismatched and non-complementary targets and it is similar to diagrams without targets (negative control). In opposite, in the presence of the complementary targets, fluorescence intensity increased, strongly. In fact, Figure 5 shows the selectivity of our proposed nanostructure where can distinguish complementary targets, easily. The results demonstrate that the DNA nanostructure could be used as a selective method for multiplex discrimination among nucleic acid targets and non-targets.

### Real sample analysis

**Figure 6:**
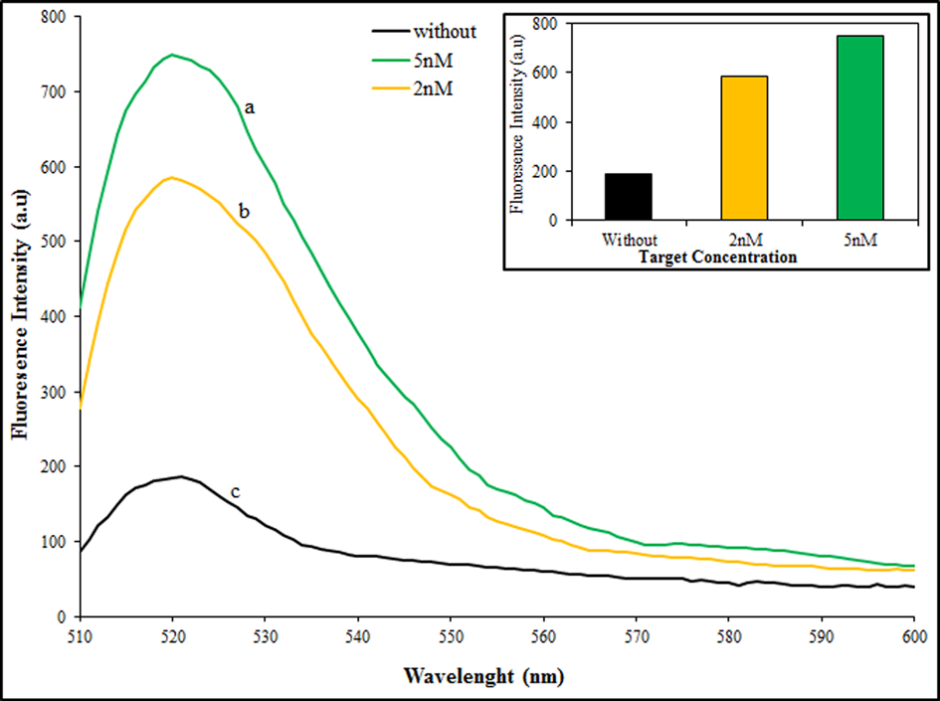
Real sample analysis Fluorescence emission spectra of HCR product in the present of target miRNAs, 5 nM (a) and 2 nM (b) of miR-137 with 50 nM of miR-142, and without targets (c) in diluted human serum. The excitation wavelength was 490 nm.

Due to the important role of early and precise diagnosis of AD for efficient therapy and to find a feasible application of the nanobiosensor in real samples for clinicians, we detected both miRNAs in human serum samples. To prepare human serum sample, the blood was taken from a healthy woman according to ethics instrumentations. Two concentrations of target miRNAs (2, 5 nM) were added to the diluted human serum. As shown in Figure 6, the results proved that the proposed method can be used in real blood samples for multiple biomarkers detection.

## Conclusions

The research on miRNAs since their discovery has been increased, exponentially, and we know that they control gene regulation that is associated with the steps of different diseases progression including Alzheimer’s pathogenesis. The discovery of miR-142 and miR-137 as biomarkers related to AD, can improve the diagnosis in its early phase and management. In this work, we have developed a label and enzyme-free ratiometric fluorescence nanobiosensor using HCR reaction for DNA amplification, SYBR green as double-stranded DNA intercalating fluorescent signal reporter and GOX as quencher of the dye fluorescence to identify and quantify the target miRNAs. Our approach is based on hybridization of the target miRNAs with three oligonucleotide probes and creation a cascade of hybridization events and is followed by nicked double helix molecule formation that eventually increases the fluorescent emission. The increase in fluorescence emission intensity is linearly proportional to the different concentrations of the target miRNAs from 0.5 to 5 nM, with less limit of detection of 0.082 nm. In contrast with frequent reported works related to miRNAs detection, the proposed method can detect two miRNA targets simultaneously and also does not require any modification of DNA probes or nanoparticle surface, and it is the first report that uses a single nanostructure with a simple design for detection of two different Alzheimer’s biomarkers (which have different expression function) in real sample (serum) and in buffer. In fact, to detect more than one biomarker at a time, it can be verified by diagnosis of the disease, more precisely and confidently. Application of SYBR green and GOX with HCR technique creates a system with high sensitivity and selectivity, which can also be used to identify even more than two miRNAs simultaneously, with some modification in hybridization probes sequence. Also according to the real range of concentrations of miRNAs in blood (from nanomolar to femtomolar values), the results on real serum samples showed that the nanobiosensor has capability to use for cheap multiplex detection of circulation miRNAs as new biomarkers for early diagnosis purposes in diseases such as MS, Alzheimer’s or else patient’s blood sample.

## Abbreviations

AD: Alzheimer’s disease
GOX: graphene oxide
miRNA: microRNA
MS: multiple sclerosis
PCR: polymerase chain reaction
